# Identifying factors affecting the selection of heads of city health centers in Iran: A phenomenological study

**DOI:** 10.1371/journal.pone.0304759

**Published:** 2024-06-06

**Authors:** Babak Rastegarimehr, Samira Raoofi, Atefeh Zahedi, Ahmad Ahmadi Teymourlouy, Mohammad Mahboubi, Jamil Sadeghifar

**Affiliations:** 1 Department of Health Services Management, School of Health Management and Information Sciences, Iran University of Medical Sciences, Tehran, Iran; 2 Department of Healthcare Management, Research Center for Evidence-Based Health Management, Maragheh University of Medical Sciences, Maragheh, Iran; 3 Department of Public Health, Asadabad School of Medical Sciences, Asadabad, Iran; 4 Department of Public Health, Abadan University of Medical Sciences, Abadan, Iran; 5 Research Center for Evidence-Based Health Management, Maragheh University of Medical Sciences, Maragheh, Iran; 6 Department of Health Management and Economics, Faculty of Health, Ilam University of Medical Sciences, Ilam, Iran; Maragheh University of Medical Sciences, ISLAMIC REPUBLIC OF IRAN

## Abstract

**Introduction:**

Managers in the health care sector have the responsibility of accomplishing objectives and guaranteeing the excellence of services. To be chosen as a manager in a health organization, individuals must possess specific qualities and skills. Examining the process of selecting and appointing managers at the highest level of service provision might offer policymakers valuable insights into the importance of considering competences when choosing and appointing leaders of health centers. Hence, this study was conducted to identify the characteristics that influence the selection process of heads of city health centers in Iran.

**Methods:**

The present study employed a qualitative and phenomenological approach, utilizing interviews performed in the year 2023. The study sample consisted of health deputy from medical sciences universities, local health network managers, and heads of city health center. The participants were selected using a purposive selection method. A total of 16 male participants were interviewed, and the interviews were then analyzed using MAXQDA-10 software using the usual content analysis method.

**Results:**

In this study, the factors affecting the selection of the heads of health care centers were classified into two general topics: individual factors and environmental factors, and eight sub-topics, including health literacy, experience, individual characteristics, communication skills, mental characteristics, legal issues, political factors, and cultural factors.

**Conclusion:**

The development of health literacy, specialized knowledge, and communication skills to coordinate and resolve organizational problems helps train competent managers. Top health system managers, who select health center managers, must understand political and cultural variables and regulate and steer their influence to select effective managers.

## Introduction

In the conditions of rapid and frequent changes that occur in various work environments, the field of primary health care is also faced with new epidemics and technological changes [1]. Healthcare organization managers must possess the ability to fulfill consumer expectations and demonstrate effective leadership in response to swift environmental changes. Hence, it is imperative to evaluate the organization’s success based on its human resources, and the management of these resources must enhance and cultivate their skills and abilities [2]. Managers in the health care sector have the responsibility of attaining objectives and guaranteeing service quality. To be chosen as a manager in a health organization, individuals must possess specific traits and talents [1].

In Iran, the health care system is structured into three levels. The city health center is situated at the second level, which serves as the primary specialized level for various health services [3]. Frequently, individuals without knowledge and proficiency in organizing and comprehending healthcare systems are selected to lead healthcare centers. The absence of transparency in the selection process and criteria for health care center managers results in the appointment of individuals lacking adequate qualifications, hence posing a challenge to the organization [4]. Several studies have demonstrated that the lack of managerial skills to effectively coordinate employee activities and strategically plan to attain health system goals leads to employee discontent and job dissatisfaction [4, 5].

In their study, Khanifar and colleagues assert that possessing knowledge and experience is a fundamental and crucial factor in the selection of competent managers [6]. In addition, researches have proposed many dimensions and criteria to assess the competencies of managers during the selection process. Occasionally, political matters have been given prominence alongside individual traits [7]. Proficiency and consciousness of contemporary technological matters are often cited as essential competences and attributes for the selection and achievement in the administration of primary health care centers [8].

Hence, to ensure the appointment of capable individuals in critical roles like health systems management, it is imperative to establish unambiguous criteria and procedures that adhere to the appropriate concepts and methodologies, hence selecting individuals with the requisite skill set. Moreover, familiarity with these criteria and processes renders them applicable in the assessment and instructional frameworks for individuals [9]. Technical and professional competencies play a crucial role in delivering health services. The selection of managers is influenced by various factors, including legal, cultural, and political considerations, as well as personality traits, psychological features, experience, and familiarity with information technology issues [10].

The management of administrative workers in the public sector has faced increasing criticism, leading to the adoption of human resource management approaches from the private sector in recent years. Public health care organizations ought to formulate their human resource management strategies and establish specific criteria for the selection of managers. An issue in health care management is the inclusion of specialist physicians in senior positions within the organization, without considering their lack of management abilities. This leads to challenges in human resource management [5, 11].

Given the intricate nature of healthcare systems and the presence of various challenges such as rising workloads, insufficient manpower, limited financial resources, and emerging diseases, it is imperative for healthcare managers to not only consistently identify the most effective problem-solving strategies and enhance service quality, but also demonstrate their leadership abilities as a fundamental aspect of their skill set [12].

Given the changing nature and development of the healthcare field, managers play a crucial role in attaining the objectives of the healthcare system. Due to recent global developments in this field, there has been a growing demand for skilled managers in the healthcare sector. Hence, it is crucial to ascertain the criteria and selection procedure for appointing the leaders of organizations that offer primary health care services, in order to ensure their accurate and effective selection and appointment. Utilizing a merit-based selection mechanism can indeed enhance the efficacy of the health system. Thus, this study was devised and executed to determine the influential aspects in the selection of the head of city health center in Iran.

## Methods

### Study design and setting

The present study was carried out using a qualitative and phenomenological approach in the year 2023. The rationale for selecting the phenomenology approach is to precisely elucidate and ascertain the phenomena as they are subjectively experienced by individuals in a particular context.

### Contributors

The study sample consisted of health deputy from medical sciences universities, managers of local healthcare networks, and heads of city health centers. The participants were chosen by the purposive sampling technique. The criteria for entering the research include: having worked for at least 2 years continuously or intermittently in the position of health deputy from medical sciences universities or local healthcare network manager, or head of a city health center; and having a real desire to actively participate in the study. The exclusion criteria included the two factors of not being satisfied with the company and not having two years of managerial experience. Based on the principle of saturation in qualitative research, a total of 16 participants, all of whom were men, participated in the study.

### Ethical approval

The present study was conducted with the permission of the ethics committee of Abadan University of Medical Sciences, with code IR.ABADANUMS.REC.1401.037. After receiving permission, data collection and interviews were conducted.

### Data collection method

In order to collect data, a semi-structured interview, which is a commonly used method in qualitative research, was employed. The interviews were carried out utilizing an interview guide that consisted of several questions that were open-ended. This guide was built using prior research and the insights of experts. In order to assess the reliability of the interview guide, three pilot interviews were carried out. Following the completion of the interviews and subsequent review, modifications were implemented to the interview guide, resulting in the creation of its definitive edition (Table 1).

**Table 1:** Interview guide for factors affecting the selection of heads of city health centers.

1. What are the criteria for appointing heads of city health centers in universities of medical sciences?
2. What is the process of appointing heads of city health centers in universities of medical sciences?
3. What are the challenges and weaknesses of the process of appointing heads of city health centers in universities of medical sciences?
4. What are the strengths of the process of appointing heads of city health centers in universities of medical sciences?

Once the participants were identified, the vice president of research at Abadan University of Medical Sciences issued an introductory letter to individuals who were eligible to carry out semi-structured interviews. Initially, the participants were provided with a comprehensive explanation of the study’s aims, followed by the acquisition of their consent by both verbal and written means. The audio file contained verifiable consent from all participants. Following the transcription of the audio recording, every participant examined and signed the interview transcript, providing informed consent to take part in the study. Participants were guaranteed the confidentiality of their personal information and were informed that their participation was optional, giving them the freedom to withdraw from the study at any time of their choosing. Subsequently, face-to-face interviews, a prevalent technique in qualitative research, were employed. The interviews were conducted by two members of the research team from April 3, 2023 to August 15, 2023.

After completing 14 interviews, the point of data saturation was achieved, and an additional two interviews were undertaken as a precautionary measure. The interviews had a mean duration of 40 to 50 minutes, and all participants were guaranteed the freedom to withdraw from the study at any time. Simultaneously, records were made during the interview.

### Data analysis

The data obtained from the semi-structured interviews was analyzed using the usual content analysis method with the assistance of MAXQDA10 software. This analysis was conducted immediately after each interview and meeting. The data analysis was conducted concurrently with the interviews. The notes were reviewed multiple times to obtain an extensive understanding of the text, followed by a meticulous line-by-line reading. The study involved extracting concepts from the statements and perspectives of the participants, which were then organized into main and general themes for classification. Initially, the data were transformed into textual format, and subsequently, the units for content analysis were determined. During the coding process, the coders reviewed and verified the outcomes of their coding. Eventually, they established the categories for the codes.

To have a more thorough comprehension of the analytical procedure, it is important to note that once the interviews were concluded, the collected data was subjected to a five-step process of examination utilizing thematic analysis. The steps involved in this process are as follows:

- Initial exposure to the text by reading the text several times to gain a general understanding of it
- Coding (recognition of concepts and themes)
- Clustering within topics
- Forming a summary table
- Writing and describing phenomena.

### Reliability and validity of the study

Four criteria, namely credibility, confirmability, dependability, and transferability, were used to assess the consistency of qualitative data [13, 14]. Thus, to verify the data, the colleagues of the study team and an external expert collaborated as required. In addition, a suitable amount of time was designated for the interviews in an atmosphere of peace. The data gathering strategy involved conducting a semi-structured interview using an interview guide. However, the researchers reviewed the extracted codes and classes. The participants were sent a summary of the interview results for their approval.

Additionally, the content analysis method was used for data analysis at beginning of the interviews, and contractual content analysis was utilized for the final analysis. To assess reliability, the initial researcher in the research team performed the coding and obtained the approval of the second and third individuals. In the domain of transferability, the acquired information was reviewed and confirmed by an external individual and a specialist in qualitative research. To enhance the consistency of the study, integration was used in the study methodology, data analysis, and data collection. Purposeful sampling enables the inclusion of individuals who possess substantial expertise on the topic being investigated.

## Results

This study involved conducting interviews with a total of 16 male participants. Out of these individuals, five had managerial positions in the local health network, seven served as health deputy, and five were in charge of the city health center. The study participants’ ages ranged from 45 to 56 years. Out of the participants, 9 had 2 years of experience in the desired role, while the remaining individuals had above 2 years (in particular, between 3 and 4 years). There were 12 participants with a doctorate in medicine, 3 with a doctorate in philosophy, and 1 with a master’s degree. The factors affecting the selection of heads of city health centers were categorized into two main issues and eight sub-issues, as described in Table 2.

**Table 2:** Factors affecting the selection of heads of city health centers.

Main themes	Sub-themes	Final codes (N*)
Individual factors	Health literacy	Academic literacy (12)
		Job knowledge (8)
		Degree of education (11)
	Experience	Having management experience (16)
		Having work experience (13)
	Individual characteristics	Having work ethic (10)
		Charismatic character (9)
	Communication skills	Having teamwork skills (10)
		Ability to negotiate (9)
		Having crisis and conflict management skills (11)
	Mental characteristics	Ability to systematic thinking (14)
		Having creativity and innovation (9)
Environmental factors	Legal issues	Employment relationship (16)
		Approval of top managers (15)
		Legal jurisdiction (16)
	Political factors	Influence of politicians (12)
		Suggestion of the local health network manager (8)
		Influence of political changes (11)
	Cultural factors	Doctors preferred (7)

*N: The number of participants who mentioned this code in the interview.

### 1. Individual factors

#### 1–1. Health literacy

Academic literacy, level of education, job knowledge, and educational qualifications are all important aspects in this sector.

*"The individual being considered must possess the necessary qualifications in terms of their university education and specialized knowledge in order to be suitable for the position they will be assigned*.*"* (M.2)

#### 1–2. Experience

When selecting an individual to manage the local health center, it is crucial to consider their expertise in the health sector and previous experience working in primary health care organizations. Health center officials can benefit from their experience in handling complex situations that necessitate effective management and specialized protocols, together with a comprehensive understanding of work procedures, in order to achieve success.

*"When the head of the health center who is selected is already familiar with the work environment and has worked*, *he is definitely more successful than someone who comes from another environment and does not know anything about the health center and its processes*.*"* (M.9)

#### 1–3. Individual characteristics

The ethical characteristics and individual dispositions of individuals have a significant impact on their ability to manage. Extroverted individuals, characterized by their responsible and decisive nature, employ distinct strategies to make progress compared to introverted individuals, which can impact their level of success. Possessing a charismatic personality to guide and skillfully carry out activities might yield positive results.

*"However*, *a boss who is well-mannered and organized*, *has a personality that attracts others*, *and is responsible for the goals of the system will definitely perform much better*.*"* (M.5)

#### 1–4. Communication skills

Two crucial abilities worth mentioning are the aptitude for collaborative work and the proficiency in negotiation, crisis management, and conflict resolution within an organization. To facilitate progress and accomplish objectives, it is imperative for each complicated manager to establish work teams and effectively coordinate their efforts. If there are disagreements or unexpected problems, he should possess the ability to negotiate disputes and handle conflicts and crises using scientific and principled negotiation techniques.

*"Usually*, *there are differences between the employees of different departments regarding the budget*, *the difficulty of work*, *and many other issues*, *and it is the responsibility of the head of that group to be able to resolve these*.*"* (M.1)

#### 1–5. Mental characteristics

Comprehending the interconnections among various components of the system and the system’s disposition are prerequisites for managers to achieve success. Often, it is necessary to apply novel and inventive approaches, utilizing creativity, in order to address challenges and attain objectives. Hence, possessing a systemic thinking capacity together with creativity and innovation is crucial for managers to effectively lead the firm and achieve success.

*"Health centers have different departments and different tasks*, *and the manager of that center should understand the relationship between the departments in general and have respect for their duties*, *knowing that they are all important*.*"* (M.16)

### 2. Environmental factors

#### 2–1. Legal issues

Having an employment relationship, the approval of top managers, and legal authority are crucial considerations in this field. When appointing managers for health centers, it is necessary to adhere to administrative regulations. The individuals in question must undergo the legal procedure to assume managerial roles, and the relevant inquiries must be conducted by responsible authorities regarding their suitability.

*"It is possible that*, *in our opinion*, *one person can be a good manager and has good scientific and personal characteristics*, *but legally*, *it is not possible to appoint that person*.*"* (M.12)

#### 2–2. Political factors

The key aspects in this domain are the participation of parallel entities and organizations, the impact of political elections, the influence of politicians, and the role of the governor. Essentially, soliciting input from external stakeholders consistently influences the choice and effectiveness of every assortment.

*If influence is not exerted directly or indirectly from outside the group*, *the choices may be different*. *Basically*, *the interference and opinions of influential political and executive people act like a double-edged sword and can be both useful and harmful*.*"* (M.13)

#### 2–3. Cultural factors

Implicit norms, such as the organizational culture, exert a profound impact on the choices and conduct of employees. The more robust this culture is, the greater its effectiveness. Within the Ministry of Health, there exists an unspoken preference for doctors over non-doctors when it comes to assuming responsibilities. The selection of sub-category managers is mostly influenced by the opinion of the highest authority. Appointments are formally established through written notification sent by the highest-ranking individual within the organization.

*"It is a fact that in the Ministry of Health*, *doctors are more preferable than others*. *If you do a survey*, *you will see that there are more senior officials in this field of health than doctors*.*"* (M.7)

## Discussion

This study aimed to determine the factors influencing the selection of heads of city health centers in two main areas: the individual context and the broader context encompassing legal, cultural, and political factors. Additionally, eight sub-topics were identified, including health literacy, experience, personal characteristics, communication skills, mental characteristics, legal issues, political factors, and cultural factors.

The participants have emphasized the importance of possessing professional knowledge, expertise, and a genuine academic degree in the subject of health when it comes to health literacy among managers. In their study on the competencies required by managers in the Ministry of Health, Vakilzadeh et al. identified professional skills as essential attributes, encompassing the capacity and proficiency in performing tasks [15].

The findings of Sokolov et al. [4] have verified this outcome. Pan and Kung have also underscored the significance of possessing professional knowledge and expertise in order to achieve success in health management [16]. Proficiency in specialized knowledge and literacy is essential for achieving success in any professional domain. In the context of health centers, it is imperative for managers to possess education and expertise in the field of health. Without such qualifications, they would be unable to identify mistakes and errors, as well as employ accurate and scientific methodologies.

This study places emphasis on the importance of have prior experience and a comprehensive employment record. Salem et al. conducted a study to examine the necessary skills for managers in primary health care. According to their statement, a crucial determinant for achieving success in health care administration is possessing managerial expertise, job experience, and a comprehensive understanding of the workflow and interrelationships within various health sectors [1]. To comprehend work relationships and task coordination among employees, a thorough understanding of the work environment is important. This matter has been underscored in multiple research studies [10, 15, 17].

Fanelli et al. conducted a study to investigate the essential indicators required by managers in health systems. They deemed individuals’ personality qualities as influential factors in selecting a management style and facilitating communication with employees [5]. In addition, Sarabi and colleagues deem the personality qualities, ethics, and behavior of managers to be influential in both leadership and management. Possessing a charming demeanor is crucial in exerting influence over the performance of employees [18]. The findings of this investigation have been verified.

The present study revealed that effective communication abilities, including the capacity to engage in negotiation, establish collaborative working groups, and handle conflict and crisis situations, are crucial factors in the process of selecting managers. According to Hamidi and colleagues, managers consider communication skills to be highly significant. Proficiency in these skills is crucial for establishing work groups and engaging in collaborative teamwork [19]. Several studies [10, 20, 21] have highlighted the importance of health organization managers’ capacity to effectively handle conflict and crises.

Mosleh et al. [21] developed a competency model for health system administrators, which includes mental traits like creativity and systemic thinking. McCann and colleagues argue that for an organization to thrive, a manager must exhibit both innovation and creativity [22]. In light of the ever-changing dynamics of the health organization environment and quick technology advancements, it appears imperative for health center managers to possess innovative and systemic thinking abilities, as well as a comprehensive awareness of the organization as a whole and the linkages within it.

The findings of the current investigation indicate that legal issues, such as verifying the qualifications of candidates, play a significant role in the selection and appointment of health center managers. Vakilzadeh and colleagues assert that adherence to legal requirements and rules is a crucial factor in the successful selection of managers [15]. Another study found that court cases are efficacious in the process of appointing and selecting managers [23]. Having well-defined policies that establish a framework for selecting managers without imposing unnecessary limitations or barriers can be beneficial for ensuring an orderly appointment process that allows for the inclusion of skilled and qualified individuals.

Sarabi et al. have highlighted the impact of political issues on managers’ performance and decision-making [18]. The impact of political figures and the implementation of their viewpoints and choices can have both beneficial and detrimental effects. However, in the majority of instances, these influences and the implementation of the viewpoints of powerful politicians are conducted with the aim of safeguarding personal and partisan interests. Political leaders in society can have a good impact by ensuring transparency and enhancing the long-term stability of financial and executive systems [24]. The present study highlights the impact of political forces and individuals in the field of politics.

In this study, it was found that cultural elements, including the preference of doctors over non-doctors, played a significant role in the process of selecting and appointing managers of health and treatment centers. The influence of cultural elements on the performance and selection of managers, the quality of health services, and organizational processes has been highlighted in numerous studies [5, 21, 25]. Organizational values have a significant impact on decision-making. These cultural values serve as a cognitive framework for decision-makers to choose work methods, processes, and personnel appointments within the business.

### Limitations and strengths

The limitation of this study was the infeasibility of conducting interviews with the presidents of University of Medical Sciences. In order to minimize its impact on the accuracy of the findings, the participants were asked to concur with the president of the city’s University of Medical Sciences.

## Conclusion

In summary, the findings of this study indicate that the selection process for city health center managers should consider a range of elements, such as individual characteristics and the legal, political, and cultural context. Considering these variables can facilitate the selection of capable and effective managers for healthcare facilities, ultimately resulting in enhanced healthcare service quality and community health outcomes. When selecting health center administrators, it is important to consider each individual’s qualifications. Developing health literacy and specialized knowledge, along with training communication skills to promote coordination and resolve organizational problems, are excellent methods for educating competent managers. The senior executives of the healthcare system, who hold the responsibility for appointing managers of healthcare institutions, must recognize and handle the political and cultural elements of the context in order to ensure the appointment of capable managers.
